# Emergence of blaNDM-1 and blaVIM producing Gram-negative bacilli in ventilator-associated pneumonia at AMR Surveillance Regional Reference Laboratory in India

**DOI:** 10.1371/journal.pone.0256308

**Published:** 2021-09-08

**Authors:** Mithlesh Kumari, Sheetal Verma, Vimala Venkatesh, Prashant Gupta, Piyush Tripathi, Avinash Agarwal, Suhail Sarwar Siddiqui, Zia Arshad, Ved Prakash

**Affiliations:** 1 Department of Microbiology, King George’s Medical University, Lucknow, Uttar Pradesh, India; 2 Department of Critical Care Medicine, King George’s Medical University, Lucknow, Uttar Pradesh, India; 3 Department of Anesthesiology & Critical Care, King George’s Medical University, Lucknow, Uttar Pradesh, India; 4 Department of Pulmonary & Critical Care Medicine, King George’s Medical University, Lucknow, Uttar Pradesh, India; Suez Canal University, EGYPT

## Abstract

**Introduction:**

Ventilator-associated pneumonia (VAP) may be a life threatening nosocomial infection encountered in intensive care units. Currently the emergence of carbapenem-resistant Gram-negative pathogens has become worrisome threat worldwide.

**Material and methods:**

Endotracheal aspirates samples were collected from patients who were under mechanical ventilation for > 48 h. The bacterial isolates were identified by MALDI-TOF-MS and antibiotic susceptibility testing performed. All carbapenem resistant isolates were tested by Modified Hodge test (MHT), modified carbapenem inactivation method (mCIM), and EDTA-CIM (eCIM) and PCR were performed to detect blaIMP, blaVIM and blaNDM producing MBL genes.

**Results:**

VAP occurred in 172/353(48.7%), 23.3% had early-onset VAP and 76.7% had late-onset VAP. Males (69.2%) were found to suffer more from VAP. Prior antibiotic therapy, CPI>6, prior surgery and tracheostomy were associated with VAP. The mortality in VAP (58.1%) contrasted with non-VAP (40%). 99/169 (58.6%) Gram-negative isolates were resistant to carbapenems. *Acinetobacter baumannii*, *Pseudomonas aeruginosa* and *Klebsiella pneumoniae* were common pathogens found in late onset VAP, whereas K. pneumoniae, *A*. *baumannii* and *Staphylococcus aureus* were common in early onset VAP. The PCR results detected blaNDM in 37/172(21.5%) and blaVIM in 30/172(17.4%); 15/172(8.7%) isolates carried both genes.

**Conclusion:**

The blaNDM-1 and blaVIM genes are the main antibiotic-resistance genes that induce resistance patterns to carbapenems in VAP, highlighting CRE strains of potential public health concern and therapeutic challenge. Diagnostic laboratories in India must get on high caution for early MBL detection as it may limit the wide dispersal of MBL genes.

## Introduction

Ventilator-associated pneumonia (VAP) is one of the life-threatening nosocomial infections in intensive care units (ICUs) worldwide and accounts for 25% of all ICU infections [1]. VAP is estimated to occur in 9–27% of all mechanically ventilated patients, with the highest risk being early in the course of hospitalization. It is associated with longer hospital-stay, prolonged antibiotic usage, and increased cost of treatment, higher morbidity and with an estimated attributable mortality of 13% [2]. VAP is usually classified as either early onset, occurring within the first four days of mechanical-ventilation (MV) or late onset, developing five or more days after initiation of MV. Successful treatment of patients with VAP is a difficult and complex undertaking [3].

Microorganisms responsible for VAP differ according to the geographic areas, duration of mechanical ventilation, antibiotic dose, ventilator days, duration of ICU stay and specific patient characteristics. A number of studies have shown that Enterobacteriaceae, non-fermenters and *Staphylococcus aureus* are causative agents of VAP [1,4]. The etiology of VAP pathogens are changing over the past years, therefore early detection of pathogens and knowledge of sensitivity patterns are very crucial for better patient outcomes [5]. There is an increasing evidence of emergence of multidrug‑resistant (MDR) pathogens due to extended spectrum beta (β)-lactamases (ESBL), AmpC β-lactamases (AmpC) or metallo-β-lactamases (MBLs) [6].

Carbapenemases include three types: class A (*bla*_KPC_, *bla*_GES_ and *bla*_IMI_), class B (*bla*_NDM_, *bla*_IMP_, *bla*_VIM_, and *bla*_SIM_) and class D (*bla*_OXA-48_ like). Recently, treatment of VAP has become challenging due to emergence of class B MBLs that have a broad range, potent carbapenemase activity and resistance to all β-lactam antibiotics but not to monobactams [7]. Development of accurate methods for the early detection of carbapenem resistant bacteria is required not only for therapy but also to monitor the spread of resistant bacteria or resistance genes in hospitals and community [8]. The multidrug resistance has been increased globally that is considered a public health threat. Several previous studies revealed the emergence of multidrug-resistant bacterial pathogens from different origins especially birds, animals, fish, and food chain which may transmitted to the human consumers resulting in severe illness [9–13].

The characterization of underlying mechanisms leading to carbapenem resistance of clinical isolates in VAP is not undertaken by most clinical microbiology laboratories for therapeutic decision-making. There is paucity of data addressing microbiological aspects particularly of MBL-producing Gram-negative bacilli among VAP patients in India. This study aimed to investigate the clinicomicrobiological profiling, antibiogram and metallo-β-lactamases (MBLs) production in VAP infections with their subsequent outcome.

## Material and methods

### Study site and design

A prospective hospital-based cross sectional study was conducted over a period of one year from June 2019 to May 2020 in the Department of Microbiology, Critical Care Unit (CCU), Pulmonary Critical Care Unit (PCCU) Critical Care Units and Trauma Ventilatory Unit (TVU) at King George’s Medical University in Lucknow, India. The bacteriology laboratory of the Microbiology department is an Antimicrobial Resistance Surveillance Regional Reference Laboratory in India.

### Ethical approval

This study was approved by the King George’s Medical University U.P., Institutional Ethics Committee (Ref. code: 97^th^ ECM II B-Thesis/P89 dated 29-07-19) and written informed consent was obtained from patients’ attendants’.

### Sample size

Existing literature from Indian studies suggests an incidence of VAP ranging from 13–42%, and is highly variable in different regions. The sample size (n) is calculated according to the formula: n = z2 X P (1-p)/d2. Where: z = 1.96 for a confidence level (α) of 95%, p = proportion (0.4) and d = 0.06.

### Study subjects

We enrolled patients based on National Healthcare Safety Network’s (NHSN) new classification definition [14] for VAP, minimum 48 h on mechanical ventilation with radiologic criteria (≥2 serial radiographs with at least one of the following: new or progressive infiltrate, consolidation or cavitation), systemic criteria with at least one of the following: fever (>38°C or >100.4°F), leukopenia (<4000 white blood cell/mm3) or leukocytosis (≥12,000 white blood cell/mm3) and for adults ≥70 years old, altered mental status with no other recognized cause) and pulmonary criteria with at least two of the following: new onset of purulent sputum, or change in character of sputum, or increased respiratory secretions, or increased suctioning requirements, worsening gas exchange (eg, desaturations, increased requirements, or increased ventilator demands), new-onset or worsening cough, or dyspnea, or tachypnea and rails or bronchial breath sounds. Patients with pneumonia prior to MV or within 48 hours of MV were excluded.

### Data collection

Detailed history, including the name, age, sex, underlying clinical condition, date of admission, history of previous hospitalization, duration of ventilation, duration of hospital-stay and demographic data of patients were obtained in a structured questionnaire format and clinical outcome of each patient was noted.

### Criteria for diagnosis of VAP

The patients who fulfilled clinical and microbiological criteria (> 10 polymorphonuclear cells/low power field and ≥ one bacterium/oil immersion field with or without the presence of intracellular bacteria on gram staining and quantitative endotracheal aspirate culture showing ≥ 10^5^ CFU/ml) were considered as confirmed cases of VAP cases.

### Identification of VAP pathogens

Endotracheal aspirate sample was collected under all aseptic precautions in a sterile universal container and sent to the laboratory within 4 h at ambient temperature. The endotracheal samples were serially diluted in sterile normal saline as 1/10, 1/100, 1/1000 and 0.01 ml of 1/1000 dilution and quantitative culture was performed on 5% sheep blood agar and MacConkey agar. After incubation at 37°C in a 5% CO_2_ incubator for 24 h, colony count was done and expressed as number of colony forming units per ml (CFU/ml). The number of CFU/ml is equal to number of colonies on agar plate × dilution factor × inoculation factor. All isolates were identified by MALDI-TOF MS (bioMérieux) and antibiotic susceptibility testing was performed and interpreted as per the Clinical and Laboratory Standards Institute (CLSI) guidelines [15].

### Antimicrobial susceptibility testing

Based on the CLSI guidelines, for the Enterobacteriaceae members and non-fermenters, the antibiotics used were amikacin (30 μg), ampicillin (10 μg), amoxicillin-clavulanate (10 μg), aztreonam (30 μg), cefepime (30 μg), ceftriaxone (30 μg), cefoxitin (30 μg), cefazolin (30 μg), ciprofloxacin (5 μg), levofloxacin (5 μg), tetracycline (30 μg), co-trimoxazole (30 μg), gentamicin (10 μg), tobramycin (10 μg), imipenem (10 μg), meropenem (10 μg), ertapenem (10 μg), piperacillin-tazobactam (10 μg). For *Pseudomonas spp*., amikacin (30 μg), aztreonam (30 μg), cefepime (30 μg), ciprofloxacin (5 μg), levofloxacin (5 μg), co-trimoxazole (30 μg), gentamicin (10 μg), tobramycin (10 μg), imipenem (10 μg), meropenem (10 μg), ertapenem (10 μg), piperacillin-tazobactam (10 μg), co-trimoxazole (30 μg) and ceftazidime (30 μg) were tested. In multidrug resistant isolates colistin MICs were tested by broth microdilution (BMD). For the Gram-positive pathogens, penicillin, erythromycin, clindamycin, penicillin B, cefoxitin (10 μg), linezolid, amikacin (10 μg), levofloxacin (5 μg), tetracycline (30 μg), co-trimoxazole (30 μg)and gentamicin (5 μg) were tested by disc diffusion method and vancomycin MICs were tested by E- strip Test. Cefoxitin (30 μg) disc was used as surrogate marker for methicillin-resistant *Staphylococcus aureus*. The carbapenem resistance was screened from meropenem and/or imipenem disc (HiMedia Laboratories, India). The quality control strains used in the study were *Escherichia coli* (ATCC 25922), *Staphylococcus aureus* (ATCC 25923) and *Pseudomonas aeruginosa* (ATCC 27853).

### Phenotypic test for detection of Metallo Beta Lactamase

#### Modified Hodge test

An inoculum of 0.5 McFarland standard of E. coli ATCC 25922 was prepared in saline and then diluted with saline up to 1:10 dilutions. MHA plate was inoculated with the above-prepared inoculum with a sterile cotton swab. Meropenem disc was applied to 10 μg at the center of the MHA plate. After that test organism was streaked with help of a sterilized wire loop in a straight line out from the center to the periphery. Plates were incubated at 37°C for 24 hours. The appearance of a cloverleaf type indentation or flattening at the intersection of the test organism and E. coli ATCC 25922 within the zone of inhibition of the carbapenem susceptibility disc is positive Modified Hodge test [16].

#### mCIM (Modified Carbapenem Inactivation Method) testing

Using a sterile inoculating loop, isolates were emulsified from a fresh cultured overnight blood agar plate in a tube containing 2 ml of tryptic soy broth (TSB). 10 μg MEM disc (BD BBL Sensi-disc susceptibility test disc) was added into the above TSB inoculum and incubated. The MEM disc was removed from the TSB inoculum with a 10 μl loop wire and then the disc was placed on the previously *E*. *coli* ATCC inoculated MHA plate. MHA plate was then incubated in for 18–24 h at 37°C. The zone diameter of 6–10 mm was considered as carbapenemase producer. All isolates that were mCIM positive were tested for eCIM test [15].

#### EDTA-modified carbapenem inactivation method

For each isolate to test eCIM, TSB tubes were prepared. 20 μl of the 0.5 M EDTA was added to the TSB tube. An increase in zone diameter (mm) ≥ 5 was considered as metallo-β-lactamase producer [15].

### DNA extraction and PCR amplification of *bla*_IMP_, *bla*_VIM_ and *bla*_NDM_ genes

DNA extraction was done by boiling method [17]. Monoplex PCR was performed to detect *bla*_IMP_, *bla*_VIM_ and *bla*_NDM_ responsible for MBL production using the primers as described in Table 1. PCR amplification was done in a DNA thermal cycler (Model-Bio-Rad C1000 Touch TM Thermal Cycler) with a final volume of 25 μl master mix consisting of 12.5 μl of 2X universal PCR master mix, 2μl of primers (5–10 μM) of each forward and reverse primers, 5.5 μl of nuclease-free water and 5 μl of DNA template. The initial denaturation temperature was at 95°C for 15 min, followed by 30 cycles of DNA denaturation at 95°C for 30 sec. The primer annealing was carried out at 59°C for 1.5 min, and primer extension was carried out at 72°C for 1.5 min. After the last cycle, a final extension step was carried out at 72°C for 10 min. After that, amplification products were electrophoresed on 1.5% agarose gel and visualized using UV transilluminator at 260 nm.

**Table 1:** Primer sequences and their amplicon sizes used in amplification of MBL genes.

Gene	Primer Sequence (5’-3’)	Product size (bp)	Ref
NDM-1F	CACCTCATGTTTGAATTCGCC	984	Kaase M et al. 2011 [18]
NDM-1R	CTCTGTCACATCGAAATCGC		
VIM-F	GATGGTGTTTGGTCGCATA	390	Poirel L et al. 2011 [19]
VIM-R	CGAATGCGCAGCACCAG		
IMP-F	GGAATAGAGTGGCTTAAYTCTC	232	Poirel L et al. 2011 [19]
IMP-R	CCAAACYACTASGTTATCT		

### Statistical analysis

Statistical analyses within the study were performed using the SPSS, Version 20 (SPSS Inc., Chicago, IL, USA). Chi-square test was used to compare categorical variables where ever applicable and *p*-value less than 0.05 were considered significant.

## Results

### Incidence and characteristics of patients with VAP

Out of the 353 MV patients, 172(48.7%) met clinical and microbiological criteria and were considered cases of VAP. 55(15.6%) samples met microbiologically criteria, but cases did not meet clinical criteria and were considered as non-VAP. Early-onset VAP was found in 40(23.3%) patients and late-onset VAP in 132(76.7%) patients. Most common affected age group was 41–60 years with mean age 46.85±18.13 and range 19–89 years. 119(69.2%) patients were male and 53(30.8%) were females and difference was statistically significant (*p* = 0.026).

### Risk factors in VAP and Non-VAP patients

On univariate analysis, VAP showed significant association with prior surgery (*p* = 0.009), CPI score >6 (p<0.001) and previous antibiotic therapy (*p*<0.001). Tracheostomy showed significant association with late-onset VAP (*p* = 0.025). The proportion of tracheostomy cases was significantly higher in late VAP as compared to early VAP (36.4% vs. 17.5%). Late VAP cases as compared to early VAP cases had significantly higher ventilation time (10.02±4.27 vs. 5.75±2.47 days) and duration of hospital stay (21.29±10.93 vs. 15.35±5.85 days). The risk factors have been summarized in Table 2.

**Table 2:** Correlation of demographic and risk factors in VAP and Non-VAP patients by univariate analysis.

SN	Variable	Total cases (n = 244)	Non-VAP (n = 57)		VAP (n = 187)		Chi-square	
1.	Mean age±SD (Range)	46.85± 18.13 (19–89)	49.25±17.93 (19–82)		46.08±18.18 (19–89)		1.132	0.259
			**n**	**%**	**n**	**%**	**χ^2^**	**‘*p*’**
2.	Age (Years)							
	18–25	35	7	12.7	28	16.3	2.101	0.717
	26–40	60	12	21.8	48	27.9		
	41–60	75	20	36.4	55	32.0		
	61–80	51	15	27.3	36	20.9		
	>80	6	1	1.8	5	2.9		
3.	Sex							
	Females	79	26	47.3	53	30.8	4.975	0.026
	Male	148	29	52.7	119	69.2		
4.	Smoking	86	17	30.9	69	40.1	1.501	0.220
5.	Alcohol	41	9	16.4	32	18.6	0.141	0.707
6.	Diabetes	30	7	12.7	23	13.4	0.015	0.902
7.	Hypertension	81	18	32.7	63	36.6	0.276	0.599
8.	Liver disease	18	6	10.9	12	7.0	0.883	0.347
9.	Lung disease	39	7	12.7	32	18.6	1.012	0.314
10.	Renal disease	22	8	14.5	14	8.1	1.954	0.162
11.	Neurological	11	2	3.6	9	5.2	0.230	0.631
12.	Surgery	109	18	32.7	91	52.9	6.799	0.009
13.	Tracheostomy	68	13	23.6	55	32.0	1.382	0.240
14.	Heart disease	13	6	10.9	7	4.1	3.611	0.057
15.	CPI^a^ Score ≥6	186	15	27.3	171	99.4	146.569	<0.001
16.	Previous Antibiotic history	62	12	6.7	50	29.1	30.421	<0.001

^a^CPI score- Clinical pulmonary infection Score.

### Causative agents of early-onset and late-onset VAP

187 isolates were recovered from 172 VAP patients; the most common pathogen was *Acinetobacter baumannii* (29.4%) followed by *Pseudomonas aeruginosa* (24.1%), *Klebsiella pneumoniae* (24.1%) and *Staphylococcus aureus* (7.5%). Most common agents in early-onset VAP cases were *K*. *pneumoniae* (36.4%), *A*. *baumannii* (20.5%), *S*. *aureus* (20.5%), and while in late-onset VAP were *A*. *baumannii* (32.2%), *P*. *aeruginosa* (29.4%), *K*. *pneumoniae* (20.3%) and *E*. *coli* (5.6%) Table 3. Of the included cases, 15 (8.7%) showed the polymicrobial growth Table 4.

**Table 3:** Proportion of pathogens isolated from early-onset and late-onset VAP patients (n = 172).

SN	Organisms	Total of isolates recovered (n = 187)	Early VAP (n = 44)		Late VAP (n = 143)	
			No.	%	No.	%
1	*Acinetobacter baumannii*	55	9	20.5	46	32.2
2	*Pseudomonas aeruginosa*	45	3	6.8	42	29.4
3	*Klebsiella pneumoniae*	45	16	36.4	29	20.3
4	*Escherichia coli*	12	4	9.1	8	5.6
5	*Proteus mirabilis*	8	0	0.0	8	5.6
6	*Pseudomonas putida*	2	0	0.0	2	1.4
7	*Enterobacter hormaechei*	1	1	2.3	0	0.0
8	*Citrobacter freundi*	1	0	0.0	1	0.7
9	*Staphylococcus aureus*	14	9	20.5	5	3.5
10	*Candida albicans*	3	2	4.5	1	0.7
11	*Candida tropicalis*	1	0	0.0	1	0.7

**Table 4:** The distribution of poly-microbial isolates from VAP patients.

S.No.	Organism	No. of isolates (n)	Percentage (%)
**1.**	*Acinetobacter baumannii + Pseudomonas aeruginosa*	4	26.6
	*Acinetobacter baumannii + K*. *pneumoniae*	4	26.6
**2.**	*Pseudomonas aeruginosa + K*. *pneumoniae*	3	20
**3.**	*Acinetobacter baumannii + Candida albicans*	2	13.2
**4.**	*Pseudomonas aeruginosa + Candida albicans*	1	6.8
**5.**	*Escherichia coli + Candida tropicalis*	1	6.8
**6.**	**Total**	15	100

### Antibiotic resistance patterns in VAP patients

Out of 169 Gram-negative isolates recovered, 144 (85.2%) were multi-drug-resistant; as they were resistant to more than three groups of antibiotics. 99/169 (58.6%) Gram-negative isolates were resistant to carbapenems. Among Gram-positive organisms, 13(92.9%) isolates were MRSA but all isolates were sensitive to linezolid and vancomycin. 21.4% isolates of *S*. *aureus* showed MIC 0.5 μg/ml and in 78.6% MIC was 1 μg/ml. The colistin MIC of the isolates ranged from 0.25–2 μg/ml and no resistance was seen. The antibiotic resistance pattern of isolated organisms has been summarized in Table 5.

**Table 5:** Antibiotic resistance pattern of bacteria isolated from VAP patients.

Drugs	*A*. *baumannii* (n = 55)	*P*. *aeruginosa* (n = 45)	*K*. *pneumoniae* (n = 45)	*E*. *coli* (n = 12*)*
Ampicillin	-	-	-	100
Amoxy-clavulanic acid	-	91.8		91.7
Amikacin	84.8	48.9	78.4	41.7
Tobramycin	80.4	75.6	88.2	33.3
Gentamycin	88	82.2	80.4	36.4
Ciprofloxacin	90.2	46.7	96.1	91.7
Levofloxacin	95.6	91.4	92.2	83.3
Aztreonam	-	50	90	75
Ceftriaxone	96.7	-	93.8	90
Cefoxitin	-	-	93.8	90
Cefazolin	-	-	100	100
Ceftazidime	-	62.1	-	-
Piperacillin-tazobactam	66.3	53.3	92.2	66.7
Imipenem	85.9	57.8	66.7	50
Meropenem	65.2	48.9	70.6	50
Ertapenem	-	-	56	40
Colistin	0	0	0	0
Tigecycline	0	0	0	0

### Performance of phenotypic methods for carbapenamase producers

**Fig 1:**
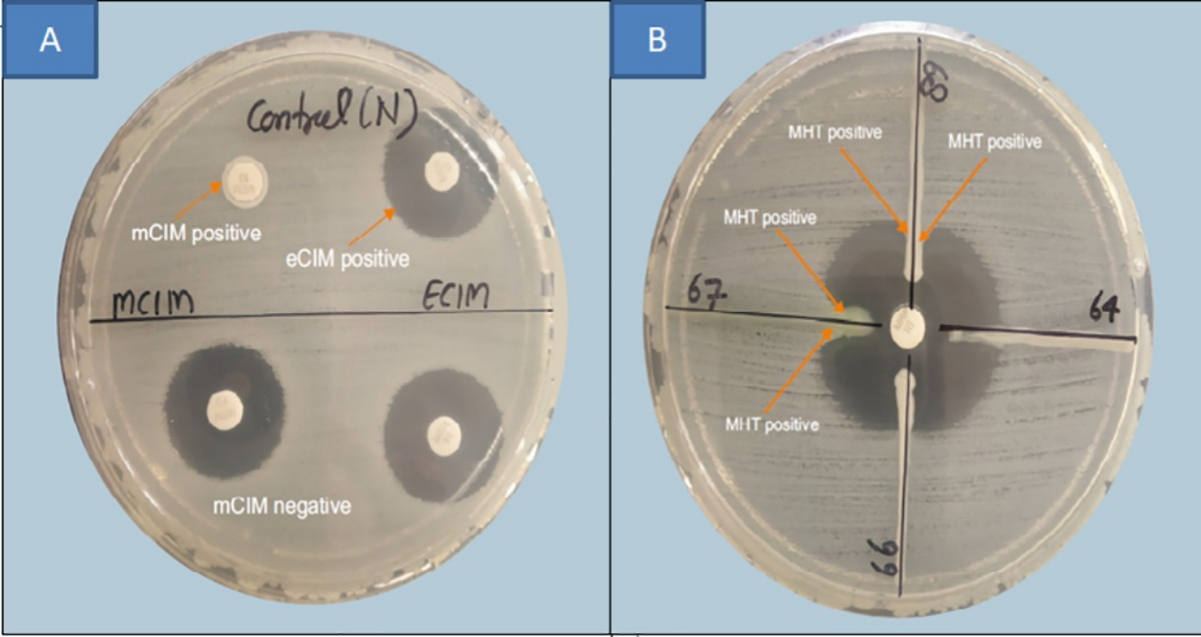
A: Isolate positive for mCIM/eCIM test (isolate with *bla*_*NDM*_ *gene)*. B: Isolate showing clover-leaf shaped pattern in Modified-Hodge test.

The phenotypic methods showed that 66/99(66.6%) carbapenem-resistant isolates were phenotypic producer of carbapenemases by the MHT while 58(75.3%) were detected by mCIM/eCIM test as MBL producers (Fig 1A and 1B). The sensitivity and specificity of the MHT was 76.9% and 44.7% respectively. The sensitivity and specificity of mCIM/eCIM test was 96.2% and 83% respectively considering PCR as the gold standard Table 6.

**Table 6:** Sensitivity and specificity of different phenotypic methods in relation to PCR among carbapenem-resistant isolates from VAP patients.

	PCR				Sensitivity	Specificity	PPV	NPV	Diag. accuracy
	Positive		Negative						
	No.	%	No.	%					
**MHT +ve**	40	76.9	26	55.3	76.9	44.7	60.6	63.6	61.6
**MHT–ve**	12	23.1	21	44.7					
**eCIM +ve**	50	96.2	8	17.0	96.2	83.0	86.2	95.1	89.9
**eCIM -ve**	2	3.8	39	83.0					
**mCIM +ve**	51	98.1	26	55.3	98.1	44.7	66.2	95.5	72.7
**mCIM -ve**	1	1.9	21	44.7					

Abbreviations: MHT, modified Hodge test; eCIM, EDTA-modified carbapenem inactivation method; mCIM, modified carbapenem inactivation method; PPV, positive predictive value; NPV, negative predictive value; VAP, ventilator-associated pneumonia; +ve-positive.

### Genotypic methods for carbapenamase producers

**Fig 2:**
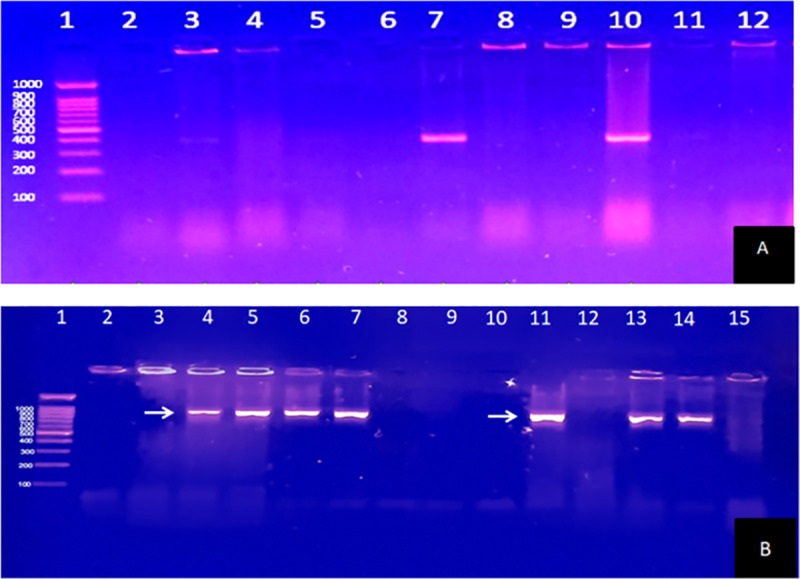
A: Agarose gel electrophoresis of products obtained by PCR of amplified DNA. Lane 1: 100 bp DNA ladder; Lane 2- Negative control; Lane 9-Positive control *bla*_*VIM*_ (390bp); Lane 10: Isolates positive for *bla*_*VIM*_ gene (390bp). B: Agarose gel electrophoresis of products obtained by PCR of amplified DNA. Lane 1: 100 bp DNA ladder; Lane 2: Negative control; Lane 4: Positive control for *bla*_*NDM*_ gene (984bp); Lane 5, 6, 7, 11,13 and 14: Isolates positive for *bla*_*NDM*_ gene (984bp).

The results of amplified genes by the PCR (Fig 2A and 2B) showed of the 99 isolates, 37/172(21.5%) contained *bla*_NDM_ and 30/172(17.4%) had *bla*_VIM_ gene. 15/172(8.7%) isolates harbored both *bla*_NDM_ and *bla*_VIM_ genes and these all were found in late-onset VAP cases. None of the isolates harbored *bla*_IMP_ gene (Table 7). NDM was more common in early-onset VAP while VIM in late-onset VAP cases. Verona integron metallo beta-lactamase (VIM) type MBL was associated with more deaths than NDM type MBL and patients with MBL negative organisms had a lesser mortality.

**Table 7:** Metallo-β-lactamases producing Gram negative isolates from VAP patients.

	Total (n = 99)	*bla*_VIM_ +ve		*bla*_NDM_ +ve		*bla*_VIM+NDM_ both +ve		*bla*_IMP_ +ve	
		No.	%	No.	%	No.	%	No.	%
*A*. *baumannii*	37	18	48.6	15	40.5	8	21.6	0	-
*P*. *aeruginosa*	24	3	12.5	5	20.8	1	4.2	0	-
*K*. *pneumoniae*	27	8	29.6	13	48.1	6	22.2	0	-
*E*. *coli*	5	0	0.0	3	60.0	0	0.0	0	-
*P*. *mirabilis*	2	1	50.0	1	50.0	0	0.0	0	-
*Pseudomonas spp*.	2	0	0.0	0	0.0	0	0.0	0	-
*Enterobacter hormaechei*	1	0	0.0	0	0.0	0	0.0	0	-
*Citrobacter freundii*	1	0	0.0	0	0.0	0	0.0	0	-
	**99**	**30**		**37**		**15**		**0**	

### Clinical outcomes

While assessing for an outcome, mortality in VAP patients (100, 58.1%) was higher compared to non-VAP (22, 34.3%). The most common cause of the death was septic shock with multisystem organ failure. 59(34.3%) patients with VAP and 31(56.4%) non-VAP patients had recovered.

## Discussion

VAP refers to pneumonia caused by bacterial agents developed in patients who are mechanically ventilated for duration of more than 48 h. It is a critical public health issue related to significant morbidity, mortality and enhanced cost of care [20].

In our study, the incidence of confirmed VAP was 48.75%. Existing literature from Indian studies suggests an incidence of VAP ranging from 13–42%, and is highly variable in different regions [21]. Incidence rate reported in the developing countries is 25–35%, while in developed countries is 15–17% [22]. Majority of patients were in age group of 40–60 years with preponderance of male sex (69.2% or 199/172), similar findings have been published in various studies [23]. It appears due to the difference in the rates of admission and enrollment. In present study, late-onset VAP (76.7%) was more common than early-onset VAP (23.3%). Few studies conducted in India showed late-onset VAP in 34–60% cases and early-onset VAP in 20–40% cases [24]. In contrast, a study conducted in Pondicherry, India observed early-onset VAP in 72.2% patients [25]. We also observed higher mortality in VAP patients 100/172(58.1%) compared to non-VAP 22/55(40%).

There are some factors that make the patients vulnerable to develop VAP. The present study identified prior surgery, CPI score >6, previous antibiotic therapy and tracheostomy were associated with VAP compared to non-VAP. The proportion of tracheostomy cases was significantly higher in late-onset VAP as compared to early-onset VAP. Patients with late-onset VAP had higher ventilation time and duration of hospital stay. Previous antibiotic treatment is a well-known risk factor for VAP [26]. However, few observational studies found antibiotic treatment to be protective against early-onset VAP [27]. The study in a Europe, demonstrated that tracheotomy was independently associated with decreased risk for VAP which is contrasting to our findings [28].

In present study, Gram-negative bacteria were found as causative agents of VAP which is similar to what has been reported in few studies [29,30]. Gram-positive bacteria are common agents of VAP in developed countries but have also been reported in few Indian studies [31]. In accordance to other Asia studies [23], multidrug-resistant *A*. *baumannii* was the predominant bacteria in VAP cases followed by *P*. *aeruginosa* (24.1%), *K*. *pneumoniae* (24.1%) and *S*. *aureus* (7.5%). Most common agents in early-onset VAP were *K*. *pneumoniae* (36.4%), *A*. *baumannii* (20.5%), *S*. *aureus* (20.5%), and while in late-onset VAP were *A*. *baumannii* (32.2%), *P aeruginosa* (29.4%), *K*. *pneumoniae* (20.3%) and *E*. *coli* (5.6%). But the probable cause for this difference could not be explained. The results of our study showed mono microbial infection in the majority of patients and 15(8.7%) patients had polymicrobial infection which can result in poor prognosis [32].

Knowledge of the susceptibility of pathogens to antimicrobial agents is urgently required, since understanding of the pattern of antibiotic resistance may aid in treatment of VAP infection. In present study, the majority of isolates from both early-onset and late-onset VAP were multidrug resistant (85.2%), carbapenem-resistant (58.6%) and resistant to typically recommended for empirical initial therapy for VAP. In the study, tigecycline and colistin showed promising efficacy followed by piperacillin/tazobactum combination and the imipenem. Among these isolate, the MIC values for colistin ranged from 0.25–2 μg/mL and for tigecycline ranged from 0.125‑2.0 μg/mL. Similar to other studies [5], we observed that MRSA and MSSA isolates were 100% sensitive to vancomycin and MIC ranged from 0.5–1 μg/ml. The incidence of MDR pathogens was quite high in our study; investigators have stated that MDR is usually a consequence of management based on empirical broad spectrum antibiotics. Thus, appropriate and judicious use of antibiotic to treat VAP, empirically, timely awareness and intervention can potentially reduce VAP and thus suffering in these patients [33]. The widespread use of over the counter antibiotics in India have led to huge selection pressure and MDR problem is likely to get substantially worse in the foreseeable future [25].

Carbapenem resistance in Gram-negative bacteria is an emerging worldwide challenge in the critical care settings. World Health Organization (WHO) in 2017 included carbapenem resistant Enterobacteriaceae (CRE), carbapenem-resistant *Pseudomonas aeruginosa*, and carbapenem-resistant *A*. *baumannii* in the highest priority category [34]. The understanding if isolate is carpapenamase producer has significant epidemiological implications for monitoring local epidemiology and also lead to more effective treatment of infections [35]. In recent years, numerous genotypic and phenotypic assays for detecting carbapenemases have been developed. The advantages of phenotypic assays compared to genotypic tests are that they are substantially less expensive than genotypic tests [36]. The overall sensitivity and specificity of mCIM test in this study was 98% and 44.6% respectively, and of MHT was 76.9% and 44.7% respectively. Our results showed that the mCIM is more accurate compared to MHT to detect MBLs. Here, we showed mCIM/eCIM had excellent sensitivity for the detection MBLs, sensitivity was 96.2% and the specificity was 83%. Our results are consistent with previous studies [35]. It is possible that new or truncated carbapenemase genes might not be identified consistently with the phenotype.

In general data on the dissemination of antimicrobial genes on India is scarce, especially regarding the prevalence of MBL genes among VAP. Worrisome, in our study is that 58.6% of VAP patients had high resistance to carbapenems. Of the 172 isolates, 21.5% exhibited the presence of *bla*_NDM_ genes and 17.4% exhibited the presence of *bla*_VIM_ gene. 8.7% isolates harbored both *bla*_NDM_ and *bla*_VIM_ genes. None of the isolates contained *bla*_IMP_ gene. Our study is in accordance to previously published ICMR report in which NDM was the most prevalent carbapenemases across the Indian AMR network [37]. In the present study, *bla*_NDM_ was harbored by15 isolates of *A*.*baumannii*, 13 *K*. *pneumoniae*, five *P*. *aeruginosa*, three E. coli and one *P*. *mirabilis*. All the isolates showed high resistance against all antibiotics, except colistin and tigecycline. In another similar study, the most common MBL subtype was *bla*_IMP_ which is contrasting to our findings [38].

*A*.*baumannii* plays a major role in VAP and acquired MBL is emerging as one of the important mechanisms of resistance [6]. In present study, 45.4% isolates of *A*. *baumannii* were MBL producers, 32.7% were VIM positive, 27.2% were NDM positive and 14.5% were positive for both. Many other studies reported higher NDM positivity (60–80%) rate among *A*. *baumannii* isolates of VAP patients [39]. In a similar study on molecular analysis showed that *A*. *baumannii* and *P*. *aeruginosa* isolates were positive for VIM gene, whereas IMP was not detected in any of the isolates [40]. In a laboratory based study, 100 MDR isolates from ICU harbored *bla*_IMP_ (89%), *bla*_VIM_(51%) and *bla*_NDM-1_(34%) [41]. There is evidence that multiples clones of metallo-beta-lactamase of *P*. *aeruginosa* are circulating in India [42]. A study from Pune, India reported VIM-type in 40% and NDM-type in 10% carbapenem-resistant *P*. *aeruginosa* isolates. This study corroborates with our findings in relation to NDM, while for VIM positivity rate is lesser than our study [43]. In the present study, *K*. *pneumoniae* harbored NDM (28.8%), VIM (17.7%) and 13.3% were positive for both VIM and NDM gene whereas *E*. *coli* only three isolates carried NDM gene. Contrary to our findings, a study from North Indian corporate hospital reported NDM gene to be more prevalent in *E*. *coli* than *K*. *pneumoniae* [37]. In present study VIM type MBL was associated with more mortality compared other MBL. The exact cause of this could not be identified. Understanding the mechanisms causing carbapenem resistance in Gram negative bacteria has important clinical implications and results in different prevention measurements and individualized antibiotic therapy. Infection control committees in hospitals should ensure robust antibiotic stewardship programs and must focus on eliminating or minimizing the incidence of VAP through preventive techniques like VAP bundle, hand hygiene, proper suctioning methods and regular fumigation of ICUs and disinfection of ventilators.

## Conclusions

To the best of our knowledge this is the first analysis of carbapenem-resistant Gram-negative bacilli carrying multiple MBL genes responsible for VAP in India. This study shows the high prevalence, diversity of patterns and coexistence of MBL genes in the Gram negative isolates from VAP patients pose risks of possible transmission to the environment, other animals and human. MBL production in VAP patients and its association with mortality is worth investigating in the future.

## Supporting information

S1 Raw images Raw images used in Figs 1 and 2. (PDF)

S1 File Data used in the manuscript. (XLSX)
