# Supplementary material for: Emergence of blaNDM-1 and blaVIM producing Gram-negative bacilli in ventilator-associated pneumonia at AMR Surveillance Regional Reference Laboratory in India
Source: PLoS One. 2021 Sep 8;16(9):e0256308. doi: 10.1371/journal.pone.0256308 (PMC8425556; doi:10.1371/journal.pone.0256308)
Supplement: S1 Raw images — (PDF) [file pone.0256308.s001.pdf]

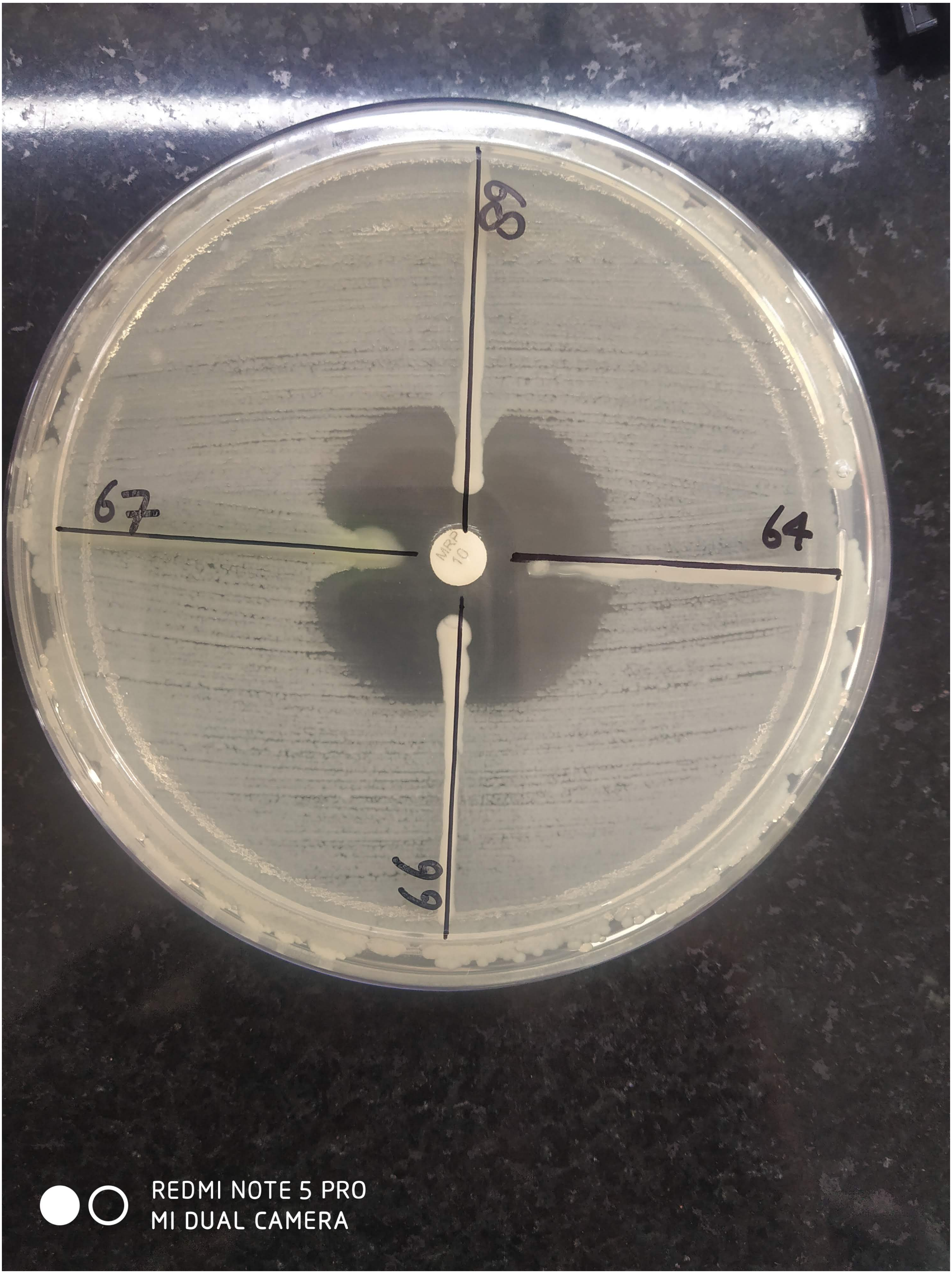
67
64
MRP
10
REDMI NOTE 5 PRO
MI DUAL CAMERA

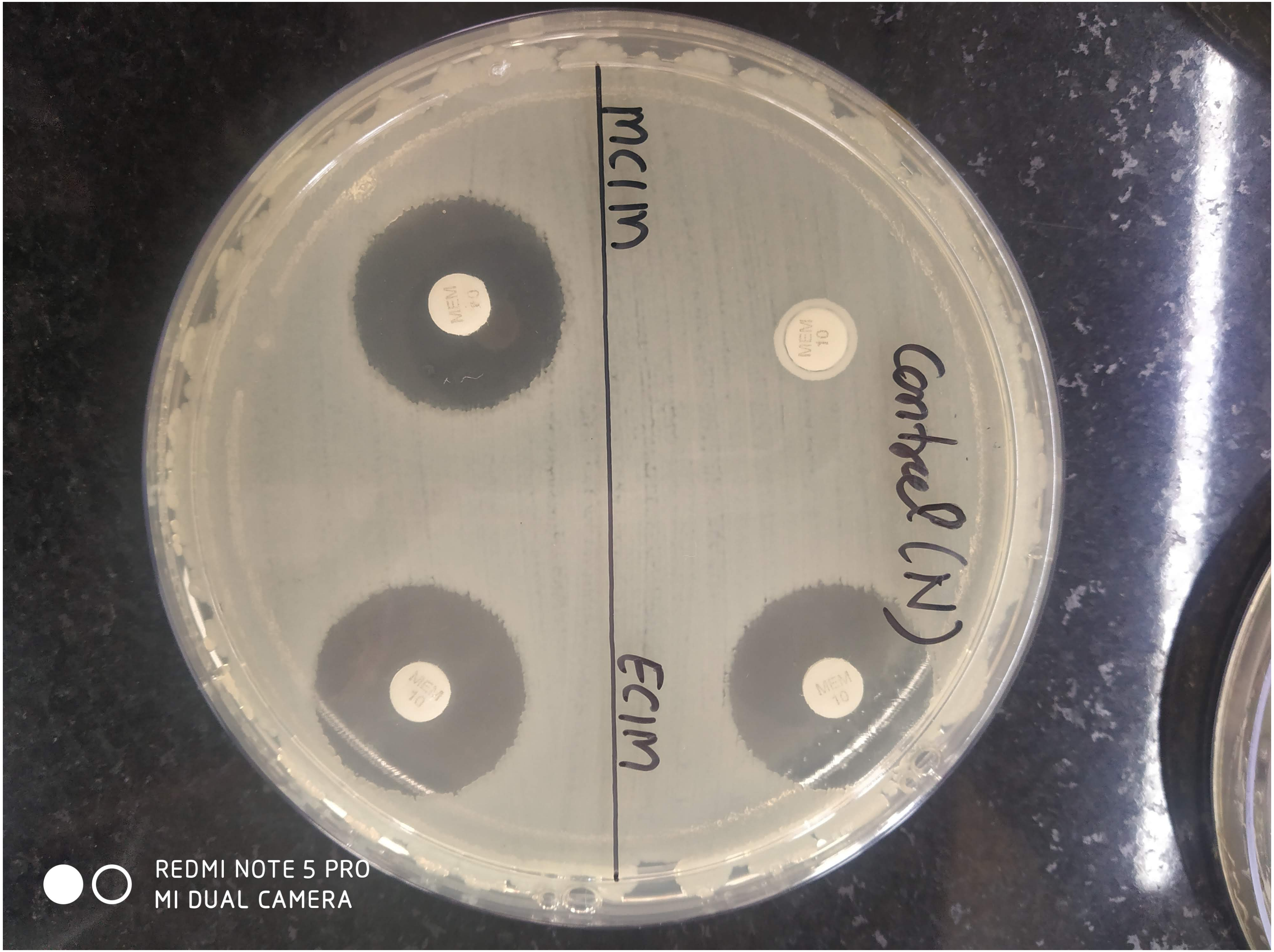
mCIM
Control (N)
eCIM
MEM 10
REDMI NOTE 5 PRO
MI DUAL CAMERA

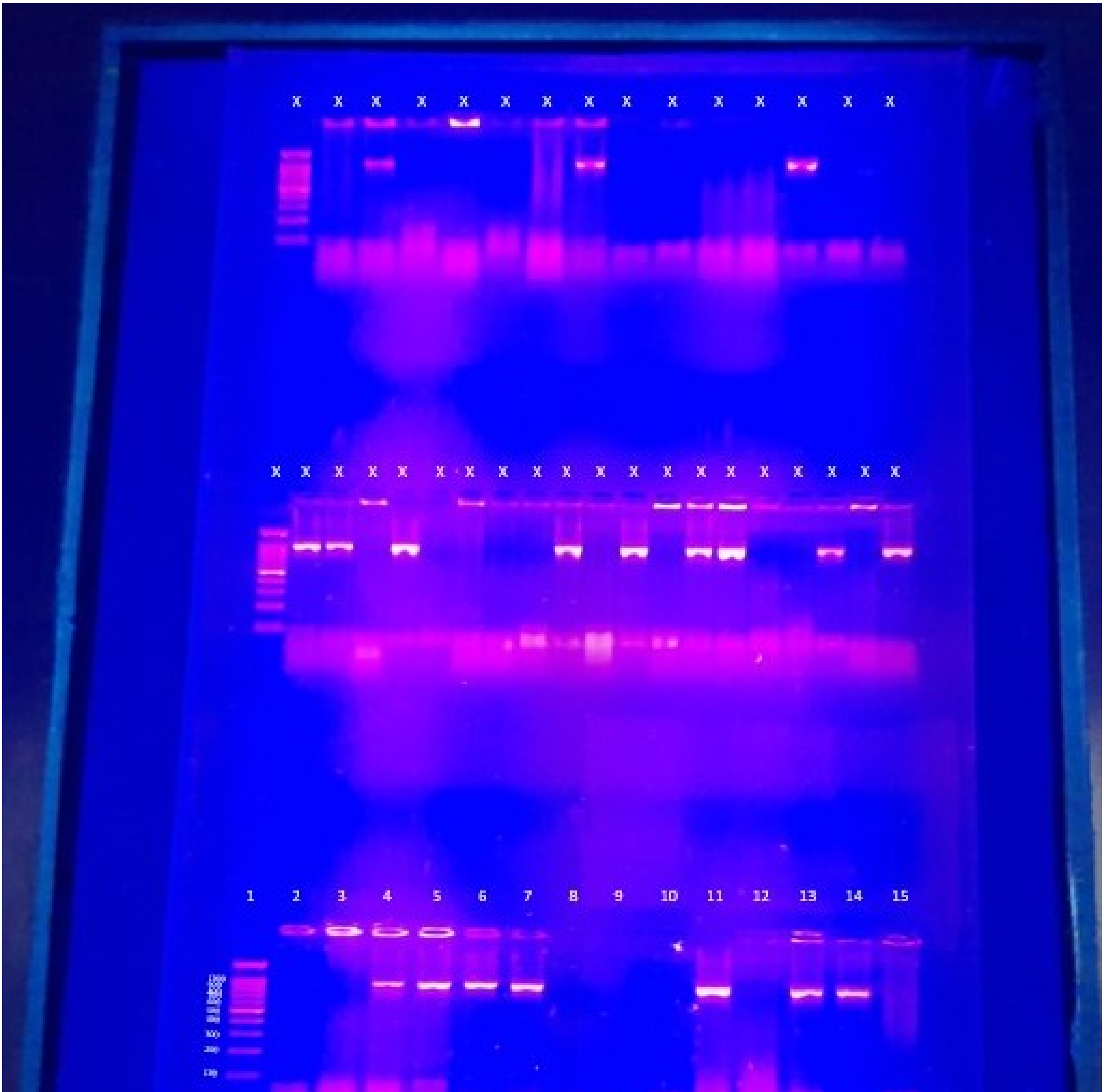

1 2 3 4 5 6 7 8 9 10 11 12 13 14 15

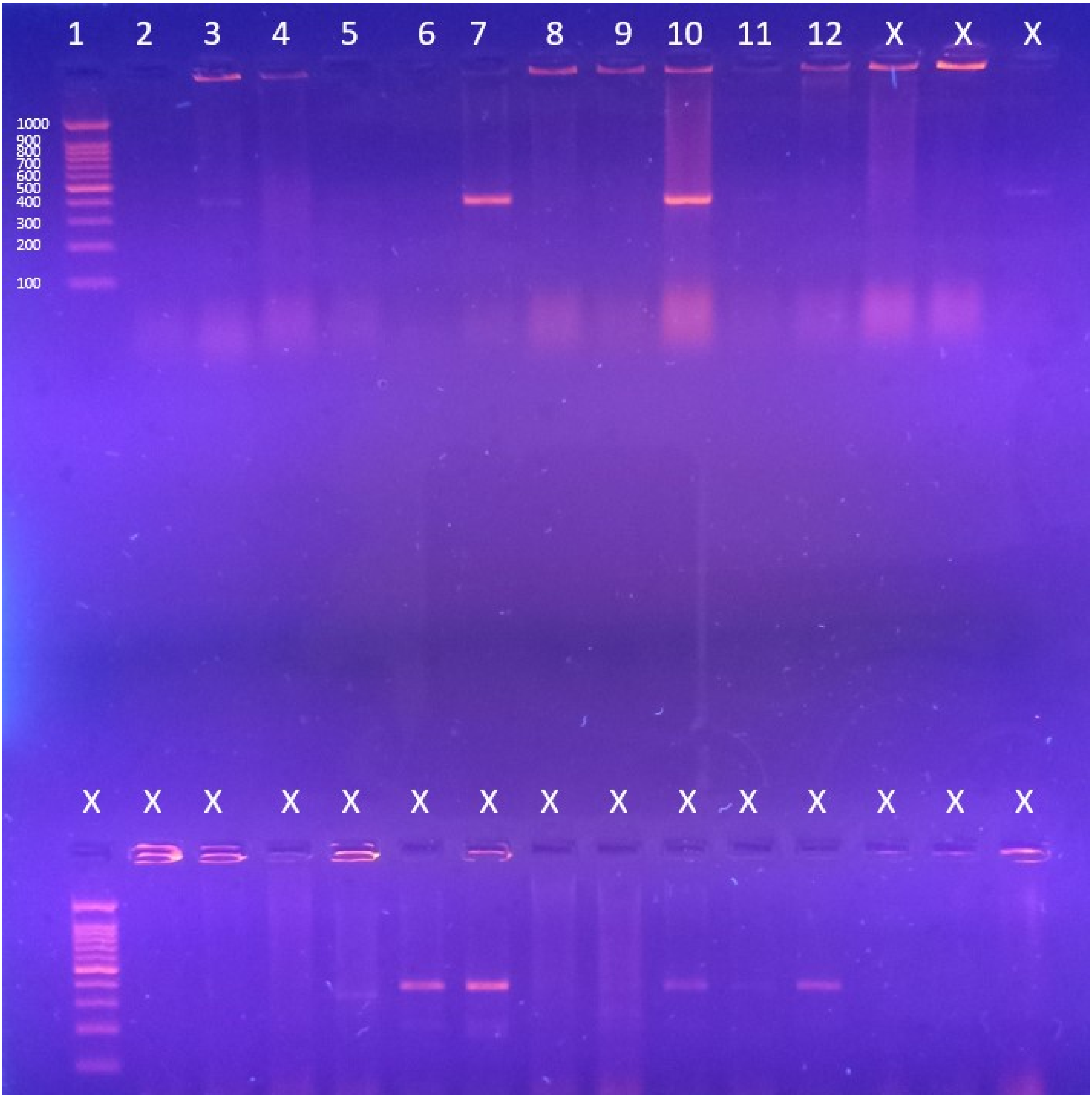
1 2 3 4 5 6 7 8 9 10 11 12 X X X
1000
900
800
700
600
500
400
300
200
100
X X X X X X X X X X X X X X X
